# Multifunctional and Flexible Phase Change Composites for Dual‐Mode Thermal Management of Lithium‐Ion Batteries

**DOI:** 10.1002/advs.202508314

**Published:** 2025-08-04

**Authors:** Lichang Lu, Haosong He, Hongxu Guo, Ignacio Martin‐Fabiani, Emiliano Bilotti, Han Zhang, Ashley Fly, Yi Liu

**Affiliations:** ^1^ Department of Materials Loughborough University Loughborough LE11 3TU UK; ^2^ Department of Aeronautical and Automotive Engineering Loughborough University Loughborough LE11 3TU UK; ^3^ Department of Aeronautics Imperial College London South Kensington London SW7 2AZ UK; ^4^ WMG University of Warwick Coventry CV4 7AL UK

**Keywords:** batteries characterisation, dual‐mode thermal management, flexible phase change composites, multifunctional composites, thermal modelling

## Abstract

Phase change materials (PCMs) are highly renowned for their substantial latent heat capacity, enabling efficient thermal management in applications such as buildings, wearable devices, and lithium‐ion batteries (LIBs). However, conventional PCMs suffer from mechanical rigidity, leakage, and low thermal conductivity. In this study, multifunctional, flexible, and leakage‐proof phase change composites (PCCs) are developed to overcome these limitations and enable dual‐mode thermal regulation for all‐climate LIBs. The PCCs provide Joule heating (22.5 °C min^−1^) under subzero conditions to prevent lithium plating and restore capacity. Simultaneously, they deliver passive cooling to optimise the operating temperature of LIBs, acrosspower output scenarios (2C and 3C). The performance is further supported and validated through COMSOL simulations, which shed light on PCCs’ phase change behaviour, the working temperature, and the heat distribution of LIBs. The integration of carbon nanofillers significantly enhances thermal conductivity by 240% while maintaining structural integrity. Additionally, the PCCs can function as overheating switches and temperature sensors (7.2%/°C at 40–45 °C) through a positive temperature coefficient (PTC) effect. Featuring low thickness (≈550 µm), leakage proof, and mechanical flexibility, these PCCs present a promising solution for advanced thermal management for safer and more efficient LIB operation.

## Graphical abstract

This study develops flexible, leakage‐proof phase change composites with dual‐mode thermal management for lithium‐ion batteries. The composites offer Joule heating, passive cooling, and temperature sensing. Simulations validate their performance and provide insights into material optimisation. Combining mechanical flexibility with intelligent thermal management, these materials are well‐suited for next‐generation batteries, flexible electronics, and wearable technologies.

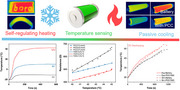

## 1 Introduction

The global energy landscape is at a critical moment, marked by rapid population growth and rising energy demands.^[^ ^1^ ^]^ In this context, lithium‐ion batteries (LIBs) have become indispensable components of energy‐efficient and sustainable technologies due to their high energy density, long cycle life, and reliability.^[^ ^2^ ^]^ However, as LIBs technology advances toward higher energy densities, flexible designs, and ultra‐fast charging capabilities, managing the significant amount of heat generated during operation becomes a critical challenge.^[^ ^3^, ^4^, ^5^ ^]^ The performance, safety, and longevity of LIBs are highly temperature‐sensitive, as thermal fluctuations can impair their active materials and electrochemical reactions.^[^ ^6^, ^7^ ^]^ Inadequate thermal management can result in catastrophic failures such as thermal runaway, fires, or explosions, posing safety risks and undermining user confidence.^[^ ^8^, ^9^, ^10^ ^]^

**Figure 1:**
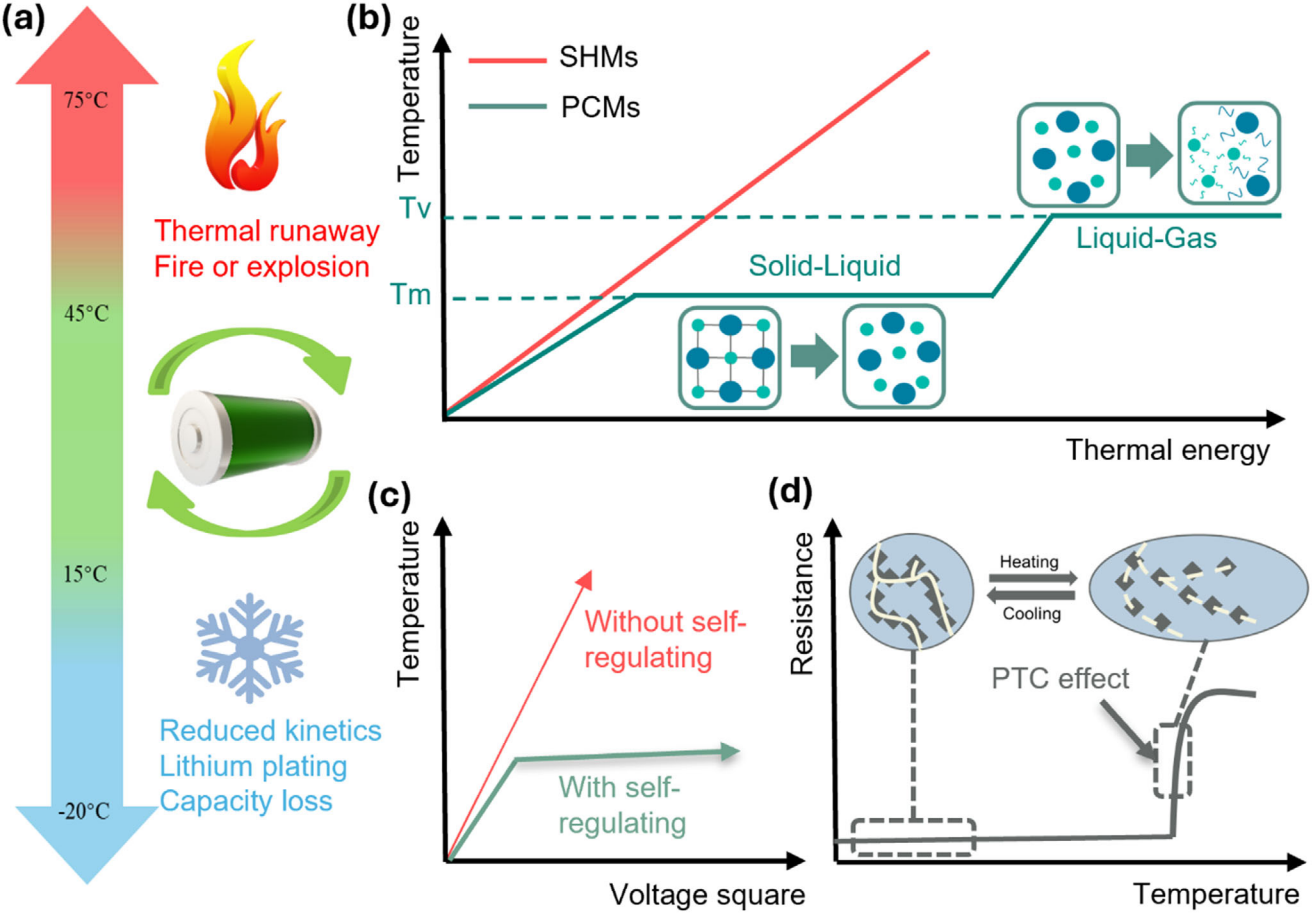
a) Critical temperatures and working conditions of lithium‐ion batteries, illustrating the risks of thermal runaway, fire, or explosion above 75 °C and capacity degradation at subzero conditions; b) Schematic representation of the working principle of PCMs in comparison with sensible heat materials (SHMs); c) A comparison diagram of the Joule heating performance with/without self‐regulation; d) The mechanism illustration of the PTC effect, with conductive filler networks showing the temperature dependence.

On the other hand, effective preheating systems are essential to prevent lithium plating and recover useable capacity under subzero conditions to ensure reliable operation, particularly in high‐latitudes regions.^[^ ^11^, ^12^, ^13^ ^]^ Therefore, developing efficient dual‐mode thermal management systems that provide both heating and cooling as required is crucial. Such thermal management system can maintain an optimal operating temperature range (15–45 °C), as illustrated in **Figure** **1**a, thereby maximising performance, safety, and lifespan.^[^ ^14^, ^15^ ^]^

Phase change materials (PCMs) have garnered significant attention for thermal management applications due to their latent heat capacity and energy efficiency.^[^ ^16^ ^]^ Unlike sensible heat materials (SHMs), such as woods and concrete, which increase surface temperature through heat absorption, PCMs absorb significant latent heat to overcome intermolecular forces during phase transitions, maintaining a stable temperature (Figure 1b).^[^ ^17^ ^]^ This latent heat capacity enables a passive temperature regulation with minimal energy consumption.^[^ ^18^, ^19^ ^]^ However, conventional solid‐liquid PCMs face several limitations. They require encapsulation to prevent leakage, exhibit low thermal conductivity, and lack the mechanical flexibility needed for advanced applications in wearable electronics, flexible devices, and soft robotics.^[^ ^20^, ^21^, ^22^, ^23^ ^]^ Improving the mechanical flexibility of PCMs not only enables better conformance to complex geometries but also enhances heat transfer efficiency by reducing interfacial thermal resistance.^[^ ^24^ ^]^

Various strategies have been explored to overcome these limitations, including nano/micro encapsulation, thermally conductive scaffold, and flexible elastomer incorporation.^[^ ^25^, ^26^, ^27^, ^28^, ^29^ ^]^ For instance, Li et al. fabricated flexible phase change composites (PCCs) via a salt‐templating method, demonstrating combined thermal management and electromagnetic interference shielding.^[^ ^30^ ^]^ However, their method involved energy‐intensive and time‐inefficient processes. Hu et al. integrated paraffin wax (PW) encapsulated separators into LIBs, mitigating internal temperature rise during short circuits, albeit this approach limited PCM loading and compromised energy density.^[^ ^31^ ^]^ Additionally, Jing et al. developed chemical crosslinked phase change films using PW, olefin block copolymer (OBC), and styrene‐ethylene‐butylene‐styrene (SEBS), exhibiting good thermal energy density, thermal stability, and elasticity.^[^ ^32^ ^]^ However, the irreversibility of the dense covalent cross‐linking bonds compromises the recyclability of such materials.

Despite such advances, current solutions fail to adequately address the dual challenges of high and low‐temperature conditions. Conventional subzero preheating strategies often rely on external heat sources and with poor efficiency.^[^ ^33^, ^34^ ^]^ Graphene nanoplatelets (GNPs) have been incorporated into OBC/PW blends by Xue et al. to capture light energy and enhance thermal conductivity of PCMs.^[^ ^28^ ^]^ Guo et al. introduced liquid metal‐enhanced PCMs to improve photo‐thermal conversion efficiency for preheating, an approach valid only in high‐light conditions.^[^ ^11^ ^]^ Inspired by the concept of all‐climate batteries,^[^ ^35^, ^36^, ^37^ ^]^ this study proposes a thin‐film PCC capable of both heating and cooling to optimise LIB working temperatures across diverse conditions.

Here, we present a novel, flexible PCC composed of biodegradable polyethylene glycol (PEG), polycaprolactone (PCL), and carbon fillers of graphene nanoplatelets (GNPs) and carbon nanotubes (CNTs). This composite is fabricated using a simple and scalable one‐step solution casting method. The PCCs provide efficient electro‐thermal energy conversion for preheating LIBs under moderate voltages, while its inherent positive temperature coefficient (PTC) effect self‐regulates the heating to prevent overheating (Figure 1c). This effect arises from the thermal expansion mismatch between conductive fillers and the polymer matrix. As illustrated in Figure 1d, filler separation occurs upon heating, disrupting electron transfer and increasing electrical resistance sharply, thereby limiting further heating.^[^ ^38^ ^]^ This unique temperature‐resistance relationship of the composites also enables it to function as a sensor, issuing hazard alerts when the operating temperature deviates from the optimal range. The inclusion of carbon fillers also enhances thermal conductivity, aiding in heat dissipation, while the high latent heat capacity of the composite limits temperature rise during both high‐ and low‐power output scenarios.

These performances are validated through comprehensive experimental analysis and COMSOL simulations. For the first time, simulations correlate the phase change behaviour with the state of charge (SOC) of the LIBs, offering insights into optimising PCM selection for various operation conditions. The biodegradability of PEG and PCL further ensures eco‐friendly thermal management, exploring the possibility of developing a sustainable battery system. In addition to the leakage‐proof and thermal conductive properties, the developed PCC exhibits multifunctionality and mechanical flexibility, broadening the application of PCMs for complex and flexible devices. More significantly, it provides a dual‐mode thermal management solution that combines self‐regulating heating, passive cooling, and temperature sensing capabilities. This integrated approach shows promises in improving the safety, performance, and lifespan of LIBs. By addressing these critical challenges in energy storage, this work contributes to the advancement of sustainable technologies and the broader adoption of LIBs.

## 2 Experimental Section

### 2.1 Materials

Polycaprolactone (PCL) granules, (>99% purity, weight‐average molecular weight: 55,000 g moL^−1^, melting point: 60 °C, glass transition temperature: −60 °C, melt flow index: 9 g 10 min^−1^ at 80 °C, 2.16 kg) were purchased from Easycomposites Co Ltd. Polyethylene glycol of two molecular masses (PEG1500: average molecular mass: 1400–1600 g moL^−1^, melting range: 43–49 °C, density: 1.2 g cm^−3^; PEG1000: average molecular mass: 950–1050 g moL^−1^, melting range: 33–40 °C, density: 1.2 g cm^−3^) were purchased from Sigma–Aldrich Co Ltd. Graphene nanoplatelets, supplied by XG Science (GNPs, grade M, 25 µm particle size, 120–150 m^2^ g^−1^ surface area, thickness: 6–8 nm, average) were purchased from Sigma–Aldrich Co Ltd. Multi‐walled carbon nanotubes (MWCNTs, formula C, diameter: 50–90 nm, 65 nm in average, length: > 6.5 µm, aspect ratio > 100) were purchased from Sigma–Aldrich Co Ltd. Chloroform (Analytical reagent grade, 99.8+%, stabilised with amylene) was purchased from Fisher Scientific Co Ltd.

### 2.2 Phase Change Composites (PCCs) Fabrication

**Figure 2:**
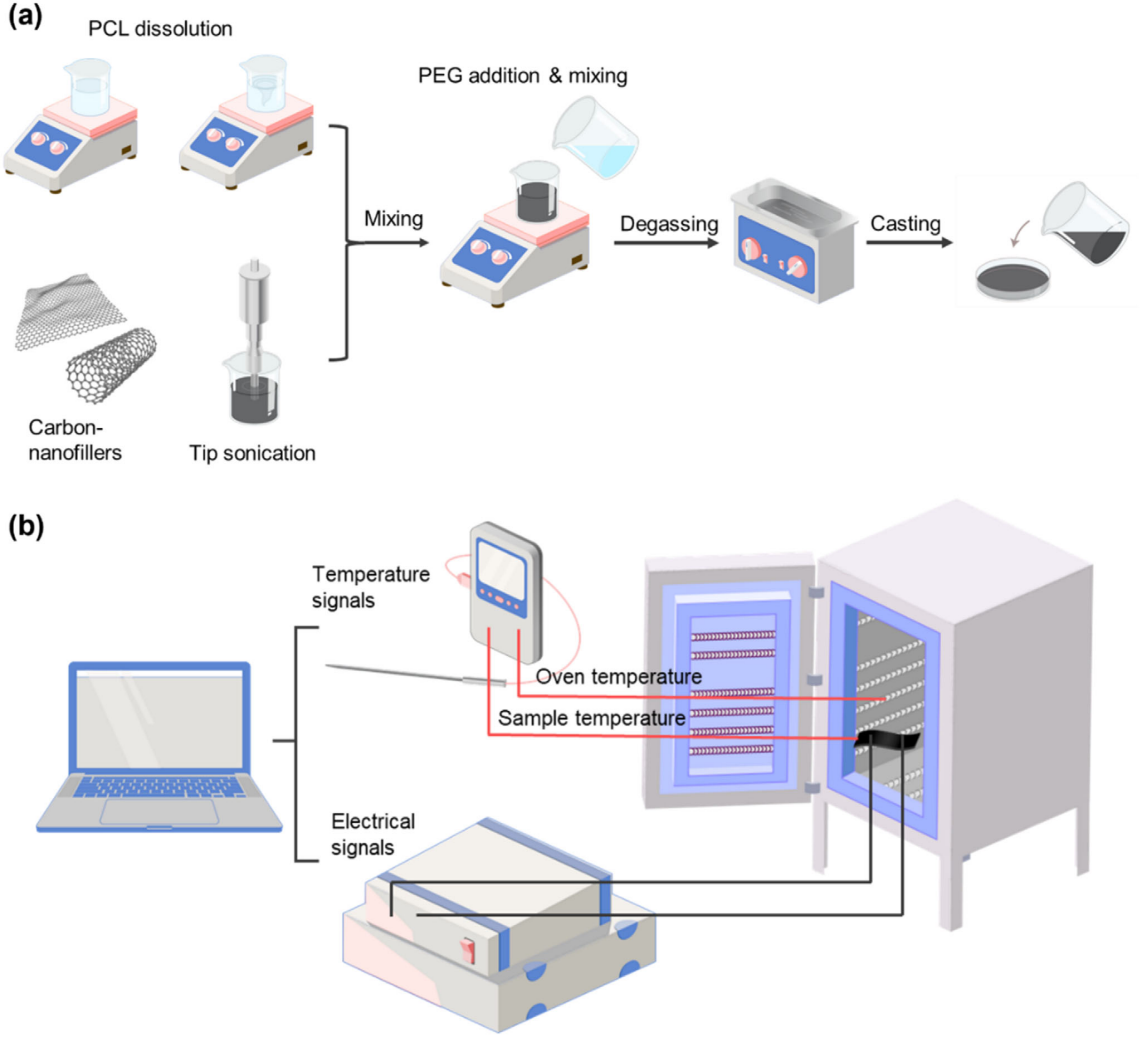
a) The Solution casting route of carbon nano‐fillers incorporated flexible phase change composites; b) The demonstrative diagram of PTC intensity measurement set‐up.

PCL was dissolved in chloroform under magnetic stirring at 500 rpm for 2 h, while GNPs and CNTs were simultaneously dispersed at a 1:1 ratio in chloroform. The dispersion of carbon nanofillers was enhanced using a tip sonicator (FS sonic dismembrator model 500) for 1 min, applying a 2 s pulse at 350 W. Subsequently, PCL solution and carbon nanofillers solution were mixed for 30 min at 1000 rpm, followed by the addition of varying amounts of PEG and stirring for 2 h at 1000 rpm. After achieving homogeneity, a 15 min bath ultrasonication was used for degassing. The resulting solution was cast onto a glass Petri dish and left to dry overnight, as shown in **Figure** **2**a. The resulting PCC films are designated as PCC(a/b)‐c% where a/b and c denote respectively the weight ratio of PCL (a) to PEG (b), and the weight percentage of carbon nanofillers (GNPs and CNTs at a constant 1:1 ratio).

After identifying the optimal ratio of PCL/PEG‐3/7 to maximise latent heat capacity (Figure S3, Supporting Information) while maintaining leakage‐proof, this ratio and the filler loading of 4 wt.% were kept constant while varying the molecular weight of PEG. Adjusting the molecular weight of PEG allows the PCCs to exhibit different melting points, enabling them to be tailored for a range of power output conditions. Further details, including various ratios and filler loadings, are provided in **Table** **1**.

**Table 1:** Phase change composites fabricated at different filler loadings and ratios.

SAMPLE	A/B – RATIO OF PCL/PEG	C% – HYBRID FILLER LOADINGS AT 1:1 RATIO	NOTE
PCC(A/B)‐C%	7/3, 5/5, and 3/7	0.3, 0.5, 0.8, 1, 1.5, 4	Optimising the ratio between PCL and PEG to balance latent heat capacity and leakage‐proof
PCC1500	3/7	4	Using optimum ratio of 3/7 and constant filler loading, varying PEG molecular weight for various conditions
PCC1000	3/7	4	

### 2.3 Characterisation

The PCCs were subjected to comprehensive characterisation to evaluate their structural, thermal, electrical properties, and thermal management performance using the following methodologies:

#### 2.3.1 Morphological Analysis

The surface and cryogenically fractured cross‐sectional area morphologies of PCCs were examined using field‐emission scanning electron microscopy (FE‐SEM, JSM‐7100F), operating at an accelerating voltage of 5.0 kV and a probe current of 8 pA. Prior to imaging, all samples were coated with a thin gold/palladium layer via sputtering to enhance conductivity.

#### 2.3.2 Leakage and Shape Stability

The leakage percentage of phase change materials was determined by calculating the weight loss before and after subjecting samples to heating at 50 °C (above the melting point of PEG) for 24 h. Subsequently, PCCs were immersed in water for 72 h for a selective removal of PEG. Shape stability was assessed by comparing digital photographs (iPhone 14 pro) of the samples before and after the same treatment.

#### 2.3.3 Electrical Properties

The electrical resistance of the PCCs was measured by using a two‐probes method using a KEYSIGHT 34465A multi‐meter. Rectangular specimens (20 mm × 10 mm × 0.6 mm) were prepared, and the silver paste was applied to the edges to ensure effective electrical contact. The resistivity ρ (Ω⋅m) was calculated using the Equation (1):

(1) $$\rho =R\times \frac{A}{L}$$

where *R* (Ω) is the electrical resistance, *A* (m^2^) is the cross‐sectional area, and *L* (m) is the length of the specimen. The conductivity (S/m) is the reciprocal of the resistivity.

#### 2.3.4 Preheating Performance

To evaluate the pre‐heating and self‐regulatory performance, PCCs were placed in a flask of liquid nitrogen and connected to an Elektro‐Automatik‐3050 B series digital bench power supply. This setup ensures a controlled voltage supply, inducing the current through the samples. Real‐time monitoring of surface temperatures was conducted using a thermocouple data logger (Pico technology TC‐08, Type K thermocouple), capturing the heating rate and equilibrium temperature. Additionally, an infrared camera (FLIR C3‐X) was used to assess the heating homogeneity across the sample surface.

#### 2.3.5 Positive Temperature Coefficient (PTC) Intensity

The PTC intensity was examined using a dedicated setup (Figure 2b) and calculated using Equation (2).^[^ ^39^ ^]^

(2) $$I_{PTC}=\log R_{m}-\log R_{0}$$

where *R_m_* (Ω) and *R*_0_ (Ω) are the maximum resistance and the room‐temperature resistance, and *I_PTC_* is determined as the disparity between them.^[^ ^40^ ^]^ Samples were subjected to a consistent heating rate of 4 °C min^−1^ provided by an oven, with the electrical resistance measured by a multi‐meter (KEYSIGHT 34465A) and surface temperature recorded by a thermocouple data logger (Pico technology TC‐08).

#### 2.3.6 Thermal Properties

The thermal conductivity tests were carried out on C‐Therm TCi using modified transient plane source (MTPS) sensor at 25 °C. The PCC cubes (20 mm × 20 mm × 20 mm) were fabricated using solution casting, shredding, and hot press. Hot press was conducted at 100 °C for 15 min.

The thermal stability and thermal decomposition temperature were characterised by thermogravimetric analysis using a TGA 550, TA instruments. Heating temperature scans ramped from 0 to 500 °C at a rate of 10 °C min^−1^.

The latent heat capacity, melting and crystallisation temperature of PCCs were characterised by differential scanning calorimetry using DSC Q200, TA instruments. Heating temperature scans ramped from 0 to 80 °C at a rate of 5 °C min^−1^.

#### 2.3.7 Thermal Management Performance

The impact of latent heat capacity on passive cooling performance was assessed by comparing the operating temperatures of both pristine LIBs and LIBs wrapped with PCCs (dimensions: 19 mm × 66 mm × 0.6 mm) under consistent discharging conditions, at 2C and 3C. The silicone thermal grease (3.9 W mK^−1^, RS Components Ltd) was evenly spread inside of the PCCs to facilitate an efficient thermal conduction. The batteries were tested on the battery holder (Fixture BF‐2A), and the discharging rate was set by an electronic load (KORAD KEL 103). Two types of 18650 cylindrical cells including Panasonic NCR PF (2900 mA, 3.7 V) and Panasonic NCR B (3350 mA, 3.7 V), were chosen as the testing battery cells.

#### 2.3.8 Thermal Model Development

The model of heat generation and working temperature of cells established by COMSOL were developed by He et al. based on a Lumped Single Particle Model (LSPM).^[^ ^41^ ^]^ The phase change distribution model was developed based on an assumption that during the phase change processes, there is no mixing between the liquid and solid phase occurring. The details of the model development can be found in (Equations S1–S30 and Tables S4–S6, Supporting Information).

## 3 Results and Discussion

### 3.1 Leakage Proof, Shape Stability and Morphological Characterisation of PCCs

**Figure 3:**
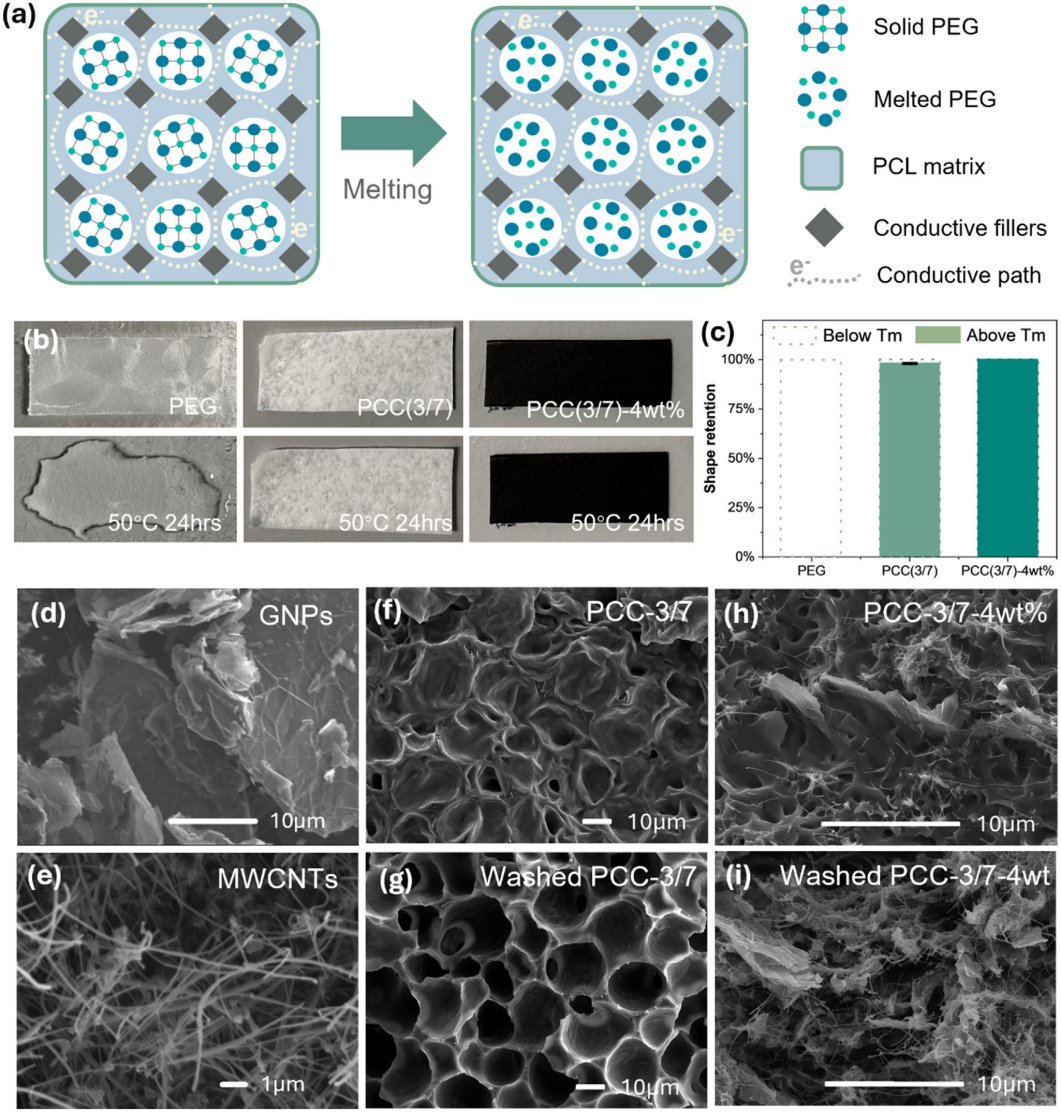
a) The illustrative diagram showing the morphology of PCCs before and after melting; b) Shape stability tests comparing PEG alone, PEG encapsulated in PCL (PCC‐3/7), and PEG encapsulated in PCL with GNP/CNT hybrid fillers (PCC‐3/7‐4wt.%), showing significant leakage reduction with the encapsulation strategy; c) Quantitative analysis of shape retention percentages for the samples after 24 h at 50 °C; d) and e) The morphology and size of GNPs and CNTs, f) and g) The comparison in morphology at micro scale between pristine and washed PCC‐3/7; h) and i) The comparison in morphology at micro scale between pristine and washed PCC‐3/7‐4 wt.%.

PEG is widely used as a phase change material due to its high latent heat capacity, tuneable melting range, and good phase change reversibility.^[^ ^16^ ^]^ However, leakage during solid‐liquid phase transitions remains a significant challenge. To address this, a biodegradable PCL matrix and a conductive filler network were used to encapsulate PEG, enabling PCCs to maintain structural integrity even above the melting point, as illustrated in **Figure** **3**a. Given PEG's melting point of 45 °C, the shape stability of PCC films was tested at 50 °C. As shown in Figure 3b, pristine PEG completely liquefies when storing at 50 °C for 24 h. In contrast, PCL‐encapsulated PEG retains its shape above the melting point, with only 2.3% of leakage, measured by weight loss (Figure 3c). An optimal PCL/PEG ratio of 3/7 maximises latent heat capacity while ensuring effective encapsulation. Additionally, the integration of a 3D conductive network constructed by GNPs and CNTs further strengthens encapsulation, achieving a leakage‐proof performance above the melting point (Figure 3c). The long‐term leakage‐proof performance is further verified under mechanical stress over ten cycles, as shown in Figure S1 (Supporting Information).

The incorporation of the hybrid filler system also addresses the inherently low thermal conductivity of PCMs. SEM imaging reveals that GNPs have a flaky structure with a high surface area, while CNTs exhibit a fibrous structure with a high aspect ratio, as shown in Figure 3d,e. The van der Waals forces between carbon fillers promote agglomeration, impairing the conductivity.^[^ ^42^ ^]^ Therefore, the tip sonication was used to exfoliate and deagglomerate the fillers. The intercalation and the synergistic effect between the flaky GNPs and fibrous CNTs form a 3D conductive network within the matrix, facilitating the collective phonon vibration and heat energy transfer.^[^ ^43^, ^44^ ^]^

Furthermore, due to the different solubility between PEG (water soluble) and PCL (organic solvent soluble), PEG can be selectively removed by immersing PCCs in distilled water.^[^ ^45^ ^]^ This selective removal assists with verifying the encapsulation efficiency on a microscale. Cryo‐fractured cross‐sectional SEM images reveal a highly porous structure in the washed sample, as shown in Figure 3f,g, confirming an effective PEG encapsulation within the polymeric network. It is worth noting that the distribution of PEG appears to be consistent across various ratios, as shown in Figure S2 (Supporting Information). This is attributed to the fabrication approach of solution casting, the partial miscibility between PEG and PCL, their asymmetric viscosity, and chain mobility. During evaporation, the phase separation between two polymers occurs, and the more viscous PCL phase results in the continuous phase, while the more mobile and smaller PEG phase tends to form droplets.^[^ ^46^, ^47^, ^48^ ^]^ A similar morphology is observed when fillers are incorporated, as shown in Figure 3h,i with a evenly‐distributed 3D conductive network within the polymer matrix, confirming encapsulation efficiency and mechanical integrity.

### 3.2 Thermal and Thermophysical Performances

**Figure 4:**
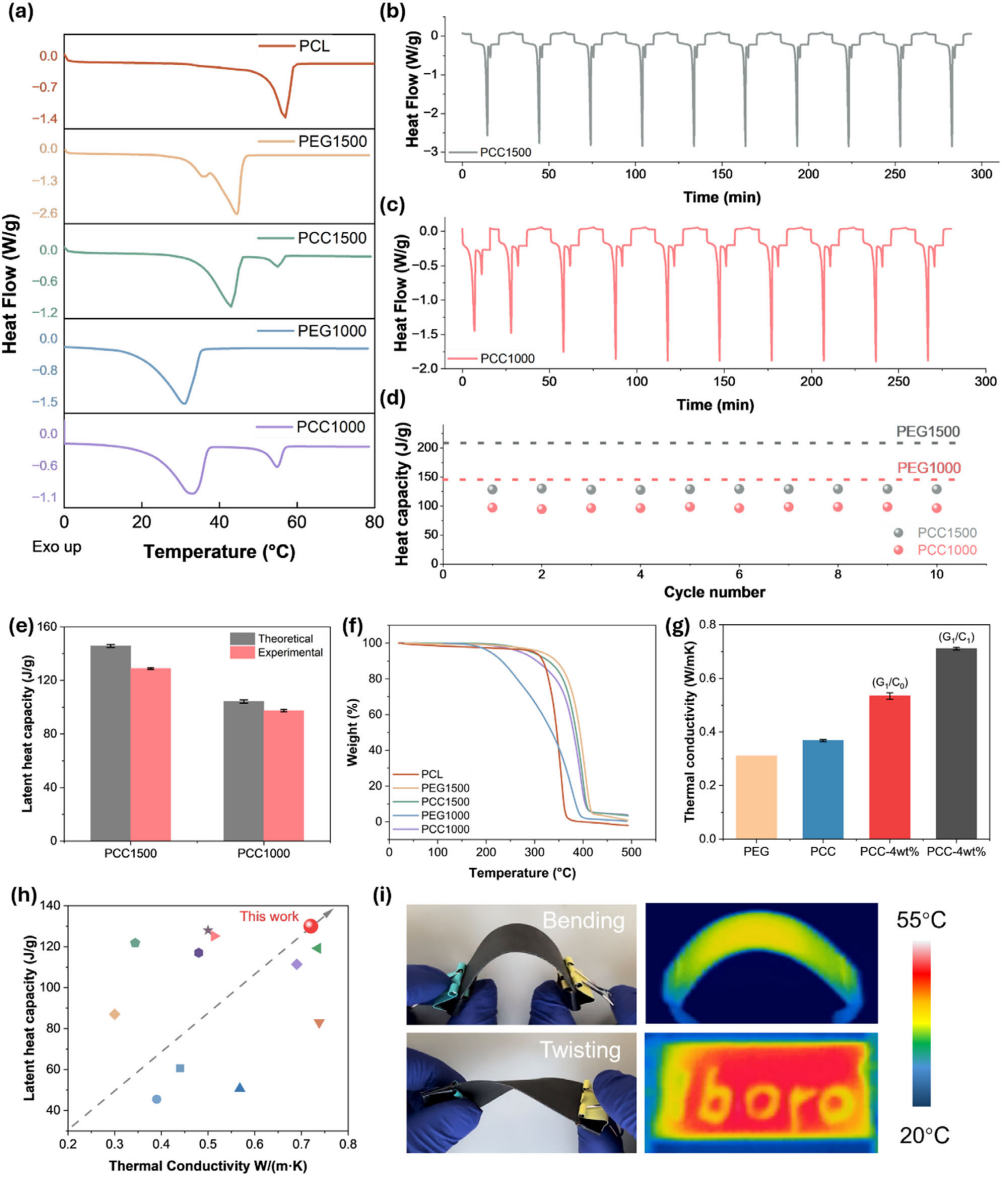
a) DSC spectrums of the PCL matrix, PEG with two molecular weights, and corresponding phase change composites; b) and c) Thermal cyclability and phase change stability of PCC1500 and PCC1000, respectively; d) Latent heat capacity retention and phase change reliability of PCC1500 and PCC1000 respectively; e) Encapsulation efficiency of PCCs, represented by the theoretical and experimental latent heat capacity; f) TGA graph of the PCL, PEG with two molecular weights, and corresponding phase change composites; g) Thermal conductivity enhancement of incorporating PCL matrix, graphene nanoplatelets, and hybrid graphene nanoplatelets & carbon nanotubes; h) Comparison of latent heat capacity and thermal conductivity between this study and other published studies, with grey arrows indicating greater performance in both properties, data resourced from ref:^[^ ^54^, ^55^, ^56^, ^57^, ^58^, ^59^, ^60^, ^61^, ^62^, ^63^, ^64^ ^]^ (from left to the right of the Figure 4h); i) Mechanical flexibility demonstration and the heating profile of the phase change composites during bending. The letters “lboro”, representing the abbreviation of Loughborough University, are handwritten using silver paste.

The key advantage of PCMs lies in their latent heat capacity, which was characterised using differential scanning calorimetry (DSC). **Figure** **4**a shows that the melting point of PCL matrix is ≈60 °C, while both PEG1500 and its corresponding phase change composite (PCC1500) exhibit a melting point near 43 °C. Notably, PCC1500 achieves a substantial latent heat capacity of 129.9 J g^−1^. Comparing the melting peaks between PEG1500 and PCC1500, the encapsulation of PEG within the PCL matrix does not affect the phase change process. The phase change point remains constant, and the latent heat capacity of the PCCs corresponds to the PEG portion. The latent heat capacity of the PCCs increases with a higher PEG content, as demonstrated in Figure S3a (Supporting Information). To achieve high latent heat capacity while maintaining a good encapsulation and leakage‐proof property, a 3:7‐PCL/PEG was selected.

For PCC1000, which uses PEG1000 with a lower molecular weight, the melting point decreases to ≈33 °C, with a latent heat capacity of 97.4 J g^−1^. The cyclability of both PCC1000 and PCC1500 was evaluated over 10 cycles, as shown in Figure 4b,c. Both composites demonstrate excellent phase change reliability and latent heat capacity retention, as shown in Figure 4d, with the pristine PEG represented by dashed line and PCCs represented by circular symbols. Detailed data, including the onset of melting process, melting point, and the latent heat capacity, are provided in Table S3 (Supporting Information). As shown in Figure 4e, according to the 3:7 ratio between PEG and PCL, both PCCs demonstrate an excellent encapsulation efficiency of 92.2% and 96.7%, calculated using Equation 3.

(3) $$\eta_{E}=\frac{\Delta H_{experimental}}{\Delta H_{Theoretical}}=\Delta H_{PCC}/\Delta H_{PEG}\times \left(1-4wt.\%\right)\times 70wt.\%\;$$

where *η*_*E*_ (%) represents the encapsulation efficiency, Δ*H_PCC_* (J/g) and Δ*H_PEG_* (J/g) represent the latent heat capacity of the fabricated phase change composites and polyethylene glycol, respectively. Additionally, (1 − 4*wt*.%) represents the weight percentage of PCL and PEG blends when minus the weight percentage of conductive fillers, and 70*wt*.% represents the weight percentage of PEG. All the values mentioned above can be retrieved from the Table S3 (Supporting Information).

Thermal stability was evaluated using thermogravimetric analysis (TGA), which measures the decomposition temperature. Figure 4f shows the decomposition temperatures of PCL, PEG1500, and PEG1000 of 330, 370, and 200 °C, respectively. When PEG was encapsulated by PCL, the decomposition temperatures increased compared to the individual components. This effect is particularly pronounced for PEG1000, where the thermal decomposition onset improves from ≈170 to ≈250 °C. This suggests a synergistic interaction between PCL and PEG, where the PCL matrix provides a protective environment by physically encapsulating the PEG and delays its thermal degradation.^[^ ^49^ ^]^ Further insights into the thermal decomposition behaviour of PCC1500 with varying PCL/PEG ratios are shown in Figure S3b (Supporting Information). The decomposition points vary slightly, and a residual 4 wt.% filler content remains after the complete decomposition of PEG and PCL.

Despite their high latent heat capacity, reversibility, and minimal supercooling, organic PCMs inherently have low thermal conductivity, limiting their ability to dissipate heat efficiently. To address this, lightweight carbon‐based reinforcement fillers were introduced. As shown in Figure 4g, the incorporation of GNPs at a low loading (4 wt.%) significantly improves the thermal conductivity by 183% compared to pure PEG,^[^ ^50^ ^]^ demonstrating the effectiveness of these fillers in improving the heat transfer. Furthermore, a hybrid filler system of GNPs and CNTs in a 1:1 ratio results in a remarkable 240% increase in thermal conductivity. This improvement is attributed to the formation of 3D conductive networks and the synergistic effect between two fillers, which helps efficient heat conduction throughout the composites.^[^ ^51^ ^]^ The merits of using a hybrid filler system are reflected in Figure S4 (Supporting Information). A considerable thermal conductivity is achieved at a low filler usage. Compared to other studies on flexible PCMs with enhanced thermal conductivity, the proposed composites achieve a balanced improvement in both latent heat capacity and thermal conductivity, as shown in Figure 4h. This synergy enhances PCC's overall thermal management performance, including efficient heat conduction and passive cooling. Additionally, owing to the subzero glass transition temperature and the flexible aliphatic backbone of PCL,^[^ ^52^, ^53^ ^]^ the fabricated PCC films demonstrate good mechanical flexibility, as shown in Figure 4i. The PCC films can bend and twist under external forces without compromising Joule heating homogeneity, highlighting their robust mechanical properties.

### 3.3 Preheating, Safety Switch, and Temperature Sensing Performances

Operating LIBs at low temperature significantly compromises their performance due to reduced electrochemical kinetics, increased electrolyte viscosity, and the risks of lithium plating.^[^ ^15^, ^65^ ^]^ These factors reduce ionic conductivity and will cause severe voltage drops during discharge, especially at higher discharging rates. The discharging C‐rate of batteries is defined using Equation (3), where *I* (A) represents the discharge current and *C_nominal_* (Ah) denotes the battery's rated capacity.

(4) $$C_{rate}=\frac{I}{C_{nominal}}$$

**Figure 5:**
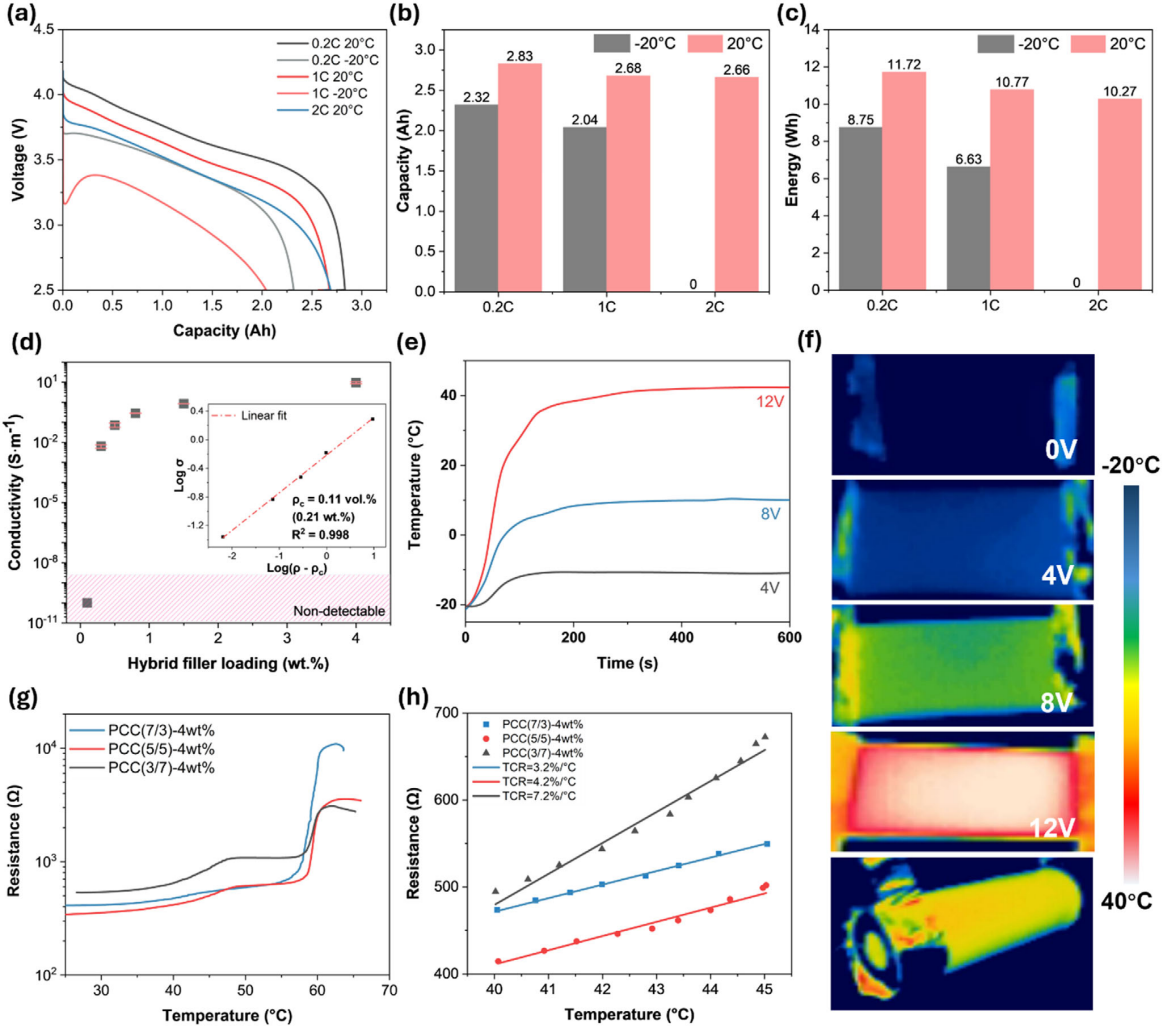
a) Discharging characteristic at various rates of the battery cell at −20 and 20 °C; b) and c) The capacity and energy at various rates of the battery at −20 and 20 °C; d) The percolation curve of the electrical conductivity of phase change composites with the incorporation of hybrid GNPs and CNTs fillers, and the fitting of the percolation; e) Joule heating capability at subzero environments under different voltages; f) The heating homogeneity of PCCs at various applied voltage; g) The self‐regulating behaviour of the phase change composites due to PTC effect; h) The temperature sensing of PCCs at the critical temperature range of 40–45 °C.

**Figure** **5**a,b, illustrates that the Panasonic NCR PF cell delivers 2.83 Ah when discharged from 4.15 V to 2.5 V at 0.2C under 20 °C. However, at −20 °C and the identical 0.2C discharge rate, the capacity drops to 2.32 Ah. This capacity reduction at subzero temperatures becomes more pronounced at higher discharge rates. For instance, at 1C discharge rate, the capacity of the battery cell drops further to 2.04 Ah. Although subzero temperatures cause a severe voltage drop, resulting in a ≈3.2 V starting voltage at 1C discharge rate, the initial discharge process generates heat, which helps mitigate this drop. As a result, the voltage gradually recovers, leading to an initial increase in the discharging characteristic curve. At 2C discharge rate, significant voltage drops caused by increased internal resistance and high current make discharging at ‐20 °C infeasible, while a capacity of 2.66 Ah is achievable at room temperature. Compared to its performance at subzero temperatures, the battery's energy output is significantly recovered at 20 °C. As shown in Figure 5c, the energy increases by 2.97 Wh at 0.2C discharge rate. At 1C, this enhancement rises to 4.14 Wh, capable of powering energy‐demanding devices. Furthermore, the LIBs can sustain operation at a higher discharge rate of 2C, retaining nearly the same energy output as at 0.2C, with a slight reduction of 1.45 Wh. The starting voltage is used for calculation to better reflect the powering capability under different temperatures.

To mitigate these challenges, electro‐thermal or photo‐thermal methods are commonly used to preheat LIBs, restoring performance and extending lifespan. Carbon‐based nanofillers, such as graphene nanoplatelets (GNPs) and carbon nanotubes (CNTs), are widely investigated for their excellent electrical and thermal conductivity.^[^ ^66^, ^67^ ^]^ Incorporating these nanofillers transforms the otherwise electrically insulating PEG and PCL into conductive composites by forming a percolative network. Figure 5d illustrates the percolation behaviour, reflecting the transition from an electrically insulating state at low filler content to a conductive state as the filler loading increases, highlighting the formation of interconnected conductive pathways. As the filler content increases from 0.1wt.% to 0.3 wt.%, the electrical conductivity of the PCCs rises dramatically from insulating to 10^−2^ S⋅m^−1^, beyond which the electrical conductivity of the composites shows only a slight increase. The percolation threshold was calculated as 0.11 vol.% (0.21 wt.%) using classic percolation theory (Equation 5).

(5) $$\sigma \;\propto {\left(\rho -\rho_{c}\right)}^{t}\;for\;\rho >\rho_{c}\;$$

where *σ* (S⋅m^−1^) represents the electrical conductivity of the phase change composites, *ρ* (vol.%), and *ρ*_*c*_ (vol.%) are the filler volume fraction and percolation threshold, respectively, and *t* serves as a critical exponent, dependent on the dimensionality of the network.^[^ ^68^, ^69^ ^]^

This equation models the relationship between filler content and electrical conductivity, fitting the experimental data to determine the critical filler concentration required to form a conductive network. At a hybrid filler loading of 4 wt.%, comprised of GNPs and CNTs at a ratio of 1:1, the PCCs achieve an electrical conductivity of 10 S⋅m^−1^, enabling efficient Joule heating capability.

This Joule heating capability remains effective even in extreme environments, such as −20 °C. As shown in Figure 5e, the PCC generates sufficient heat through electro‐thermal energy conversion, achieving a heating rate of 22.5 °C min^−1^ at 12 V. Furthermore, the heating profiles in Figure 5f demonstrate uniform heat distribution across the PCCs, minimising localised overheating and ensuring consistent temperature homogeneity.

Despite PCC's preheating ability to enhance LIB performance in extreme subzero conditions, the temperature‐sensitive nature of the battery's active materials necessitates a safety mechanism to regulate the heating process. Leveraging the positive temperature coefficient (PTC) effect, PCCs can enable an autonomously regulated heating to prevent overheating and ensure uniform heat distribution, enhancing both safety and operational reliability.^[^ ^70^ ^]^ This effect arises from the mismatch in thermal expansion coefficients between the conductive fillers (GNPs and CNTs) and the PCL matrix. Upon heating, the polymer matrix thermally expands whereas conductive fillers exhibit minimal dimensional change. This mismatch disrupts the continuity of the conductive network, leading to its breakdown and a sharp increase in electrical resistance, thereby regulating the Joule heating process even at further increased voltages.^[^ ^39^, ^71^, ^72^ ^]^ At 60 °C, corresponding to the melting point (*T_m_*) of PCL and below the thermal runaway threshold (75 °C) of LIBs, the PCC's resistance sharply increases. As shown in Figure 5g, the 7/3, 5/5, and 3/7 ratios of PCL/PEG exhibit 1.43, 1.02, and 0.76 orders of magnitude changes in resistance, respectively. This rise in resistance reduces the current flow through the material, thereby lowering the heat generated and effectively regulating the Joule heating process.^[^ ^73^ ^]^

Although the self‐regulating preheating capability helps mitigate cold condition issues in LIBs, high‐temperature risks like thermal runaway can lead to serious consequences. Therefore, issuing early hazard alerts when LIBs are approaching a risky operation temperature is of great importance. Due to the semicrystalline nature of PEG1500, the thermal expansion during phase change process contributes to the PTC effect by separating conductive fillers, thereby increasing the resistance. As shown in Figure 5h, when the LIB's operating temperature of LIBs approaches a critical threshold (40–45 °C), the PCCs can function as a temperature sensor to issue an early warning with high sensitivity (7.2%/°C). The sensitivity is reflected by the temperature coefficient of resistance (TCR), calculated using (Equation 6) ^[^ ^24^ ^]^

(6) $$TCR=\frac{R_{T}-R_{0}}{R_{0}}\frac{1}{\Delta T}\times 100\%$$

where *R_T_* (Ω) and *R*_0_ (Ω) are the resistances at the measured temperature and the ambient temperature, respectively, and Δ*T* (°C) is the temperature change. This highlights the PCC's multifunctionality, combining thermal management with safety monitoring capabilities.

### 3.4 Passive Cooling and Phase Change Behaviours at Various Power Scenarios

**Figure 6:**
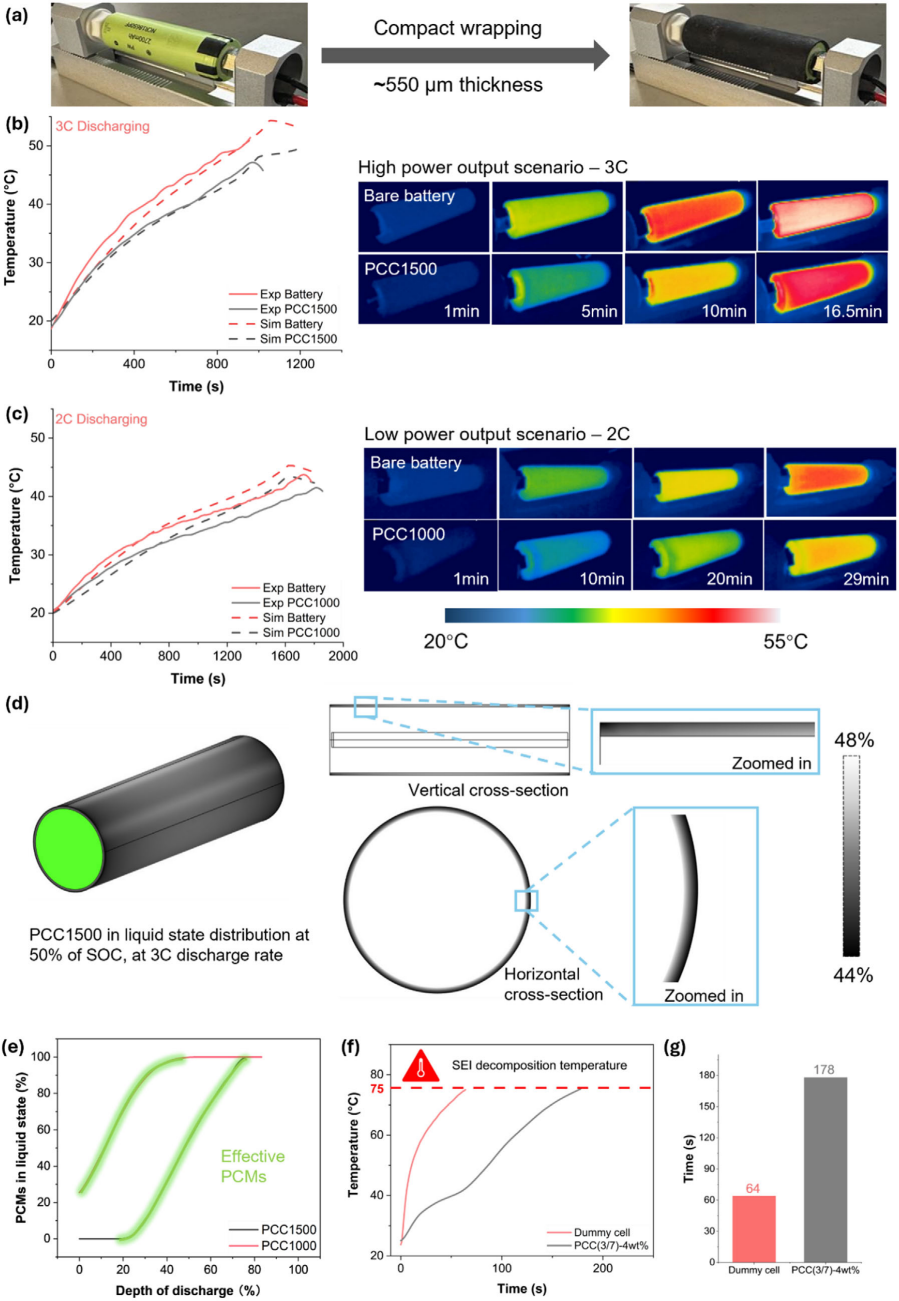
a) Experimental setup for comparing the operation temperature of battery cells with and without PCCs; b) At high power output scenario (3C), the passive cooling performance of PCC1500 to deal with the internal heat, corresponding to the thermal images; c) At low power output (2C), the passive cooling performance of PCC1000 to deal with the internal heat, corresponding to the thermal images; d) At 3C discharge rate, the phase change distribution of melted PCC1500 throughout the battery cell with the vertical and horizontal cross‐sectional images; e) The correlation between the percentage of melted PCMs and depth of discharge of batteries; f) and g) The delaying effect in temperature rise of PCCs to deal with the external heat.

The DSC analysis of the PCCs has revealed a high latent heat capacity and excellent cyclability. To evaluate its practical implications, an experimental setup (**Figure** **6**a) was designed to assess the thermal management performance of PCCs applied to LIBs under high‐rate discharge conditions, where significant heat is generated. The tight wrapping and intimate contact between batteries and low‐thickness PCCs highlight the mechanical conformability of PCCs. As shown in Figure 6b, without thermal management, the battery's surface temperature exceeds 45 °C within 659 s during high‐power operation (3C discharging condition), accelerating aging and increasing operational risks.^[^ ^74^ ^]^ In contrast, wrapping with a ≈550 µm thick PCC1500 layer lowers the temperature by ≈5 °C and maintains a plateau at the *T_m_* of PEG1500, effectively delaying the battery from reaching 45 °C by 222 s. This passive cooling effect reduces operating temperatures, extending lifespan and mitigating thermal risks. The cooling performance of PCC1500 is consistent across different battery models, such as the Panasonic NCR B with a higher nominal capacity (Figure S5a, Supporting Information). In this case, the working temperature is reduced by ≈7 °C at a 2C discharge rate, effectively preventing thermal runaway. While bare Panasonic NCR B cell reaches a risky temperature (above 45 °C), the PCC1500 employed cell maintains a lower operation temperature (below 40 °C) and a stable discharging process. Corresponding thermal images in Figure S5a (Supporting Information) validate the passive cooling performance, highlighting the temperature differences of LIBs with and without PCCs throughout the discharge process.

At a lower discharge rate of 2C, the heat generation was insufficient to trigger PEG1500 melting and utilise its latent heat capacity, as shown in Figure S5b (Supporting Information). The cooling performance of PCC1500 at 2C discharging condition is negligible. To address this, PEG1000, with a lower molecular weight and melting point, was processed using the same methodology. As shown in Figure 6c, PCC1000 achieves a similar temperature buffering effect of ≈3 °C, demonstrating the adaptability of the processing technique, tuneable melting point, and versatility of the PCCs for various power output scenarios.

To further understand PCCs performance, a COMSOL‐based heat model was developed to simulate LIB operating temperature with and without PCC application. The simulation results aligned well with experimental data, validating the robustness of the model. As shown in Figure 6d, at 3C discharge rate, PCC1500 at the centre of LIB surface, where the thermal transfer distances are the shortest, exhibits the highest temperature. Therefore, the highest extent of melting occurs, with ≈46% of PEG1500 transitioning to the liquid phase at a state of charge (SOC) of 50%. Zoomed‐in vertical and horizontal cross‐sectional images reveal a gradient melting profile, with the extent of melting decreasing progressively with distance from the centre. This phase change distribution highlights the localised thermal response of PCCs, influenced by spatial variations in heat generation and transfer within the LIBs. More importantly, the simulation established a relationship between the phase change process and the SOC of the batteries. As shown in Figure 6e, ≈25% of PCC1000 is already melted at the beginning of discharge, with complete melting occurring at ≈50% of SOC (see Figure S6, Supporting Information). This indicates that a quarter of PCC1000 is at liquid state at ambient temperature, suggesting that the onset melting may be too low. Thermal management performance is strongly influenced by environmental conditions. For instance, when the ambient temperature approaches or exceeds the PCC1000's the onset melting point, the phase change material begins to melt prematurely, reducing its capacity to absorb heat during battery discharge. Conversely, at lower ambient temperatures, the material remains in the solid state, preserving its full latent heat absorption capacity for when thermal regulation is needed. The correlation between SOC and phase change behaviour also reveals that PCC1000 provides effective thermal management only during the early stages of battery discharge. Above 50% SOC, the heat generated begins to exceed the latent heat capacity of PCC1000. This highlights an opportunity to further enhance performance by increasing film thickness or selecting materials with higher latent heat capacity or temperature. A thicker PCC layer can offer extended duration of thermal regulation, supporting safer and more stable battery operation. However, it may also introduce thermal lag and limit heat dissipation efficiency, particularly if the composites have low thermal conductivity. Tailoring the PCC thickness enables flexibility in balancing thermal performance, mechanical properties, and integration constraints, making the system adaptable to a wide range of practical use cases. Compared to PCC1000, PCC1500 provides consistent passive cooling throughout the discharge process, with complete melting occurring near the end of discharging. It also explains why PCC1500 outperforms PCC1000 in passive cooling performance. This simulated coupling provides deeper insights beyond experimental data, enabling the determination of the optimal selection of PCMs. It also expands the understanding of latent heat capacity, phase change point, and the thermophysical behaviour of the PCCs, contributing to more efficient thermal management of LIBs.

The thermal management performances discussed above addresses the abnormal heat generated by LIBs. However, elevated ambient temperatures can also pose significant risks to LIBs. To simulate this scenario, a heat gun was applied on aluminium dummy cells, mimicking rapid ambient temperature rises. Within a short period, the working temperature of LIBs reaches 75 °C, the typical decomposition temperature of the solid electrolyte interface (SEI). This rise can further melt the separator, causing internal short circuit and triggering thermal runaway. However, the addition of a thin PCC layer significantly delayed the temperature rise by 114 s, as shown in Figure 6f,g, with a temperature plateau occurring at the melting point of PEG1500. This delay provides critical time for users to take preventive actions, avoiding serial reactions and the severe consequences of thermal runaway. By demonstrating the ability of PCCs to mitigate both external and internal temperature rises, they significantly enhance the safety and reliability of LIBs, thereby boosting user confidence in their performance under abnormal conditions.

## 4 Conclusion

This study presents the development of flexible PCCs as an innovative solution for the dual‐mode thermal management of LIBs. By using simple solution mixing and casting method to encapsulate biodegradable PCMs (i.e. PEG) into polymer matrix (i.e. PCL), the PCCs effectively prevent PEG leakage while maintaining mechanical flexibility and achieving high encapsulation efficiency. The incorporation of conductive fillers, including GNPs and CNTs, enhances the thermal and electrical conductivity of the PCCs. It also enables PCCs to exhibit multifunctional properties, including efficient electro‐thermal conversion, over‐current switch, and temperature sensing. These capabilities support Joule heating in subzero conditions to regain the capacity and energy of LIBs. The self‐regulating property, driven by its intrinsic PTC effect, prevents overheating and ensures uniform temperature distribution.^[^ ^72^, ^75^ ^]^ Furthermore, the temperature‐resistance relationship allows the PCCs to function as temperature sensors, issuing early warnings at risky operating conditions. The high latent heat capacity of PCCs effectively reduces LIBs operating temperatures across diverse power output conditions, mitigating aging, preventing thermal failure, and enhancing overall safety. These passive cooling capabilities were validated through strong agreement between experimental and simulation results. Additionally, this study introduces a model that integrates phase change behaviours with the state of charge of batteries, paving the way for selecting optimum PCMs across diverse electronic applications with varying power requirements. Overall, this research advances the PCC design for dual‐mode thermal management, deepens the understanding of phase change dynamics, and contributes to safer LIB operation within the framework of sustainable energy storage technologies.

## Conflict of Interest

The authors declare no conflict of interest.

## Author Contributions

L.L. performed conceptualisation, methodology, wrote the original draft, wrote reviewed and edited the final manuscript, investigation, formal analysis, and data curation. H.H. performed methodology, investigation, wrote, reviewed, and edited the final manuscript, formal analysis. H.G. performed investigation, formal analysis. I.M.‐F. performed supervision, wrote, reviewed, and edited the final manuscript. E.B. performed wrote, reviewed, and edited the final manuscript. H.Z. performed conceptualisation, wrote, reviewed, and edited the final manuscript, methodology, and funding acquisition. A.F. performed methodology, wrote, reviewed, and edited the final manuscript, investigation, and formal analysis. Y.L. performed conceptualisation, wrote, reviewed, and edited the final manuscript, methodology, resources, supervision, and funding acquisition.

## Supporting information



Supporting Information

## Data Availability

The data that support the findings of this study are available from the corresponding author upon reasonable request.
