# Supplementary material for: Multifunctional and Flexible Phase Change Composites for Dual‐Mode Thermal Management of Lithium‐Ion Batteries
Source: Adv Sci (Weinh). 2025 Aug 4;12(40):e08314. doi: 10.1002/advs.202508314 (PMC12561345; doi:10.1002/advs.202508314)
Supplement: Supplementary file 1 — Supporting Information [file ADVS-12-e08314-s001.docx]

Supporting Information for

**Multifunctional and Flexible Phase Change Composites for Dual-mode Thermal Management of Lithium-ion Batteries**

Lichang Lu ^a^, Haosong He ^b^, Hongxu Guo ^a^, Ignacio Martin-Fabiani ^a^, Emiliano Bilotti ^c^, Han Zhang ^d^, Ashley Fly ^b^, Yi Liu ^a *^

^a^ Department of Materials, Loughborough University, Loughborough LE11 3TU, UK

^b^ Department of Automotive Engineering, Loughborough University, Loughborough, LE11 3TU, UK

^c^ Department of Aeronautics, Imperial College London, South Kensington, London, SW7 2AZ, UK

^d^ WMG, University of Warwick, Coventry, CV4 7AL, UK

^*^ Corresponding author: Yi Liu. Email: [Y.Liu2@lboro.ac.uk](mailto:Y.Liu2@lboro.ac.uk)

**Supplementary Tables and Figures**

**Table S1** Nomenclature

| $\eta_{E}$  ${\Delta H}_{PCC}$  ${\Delta H}_{PEG}$  $\sigma$  $\rho$  $\rho_{c}$  $t$ | Encapsulation efficiency  Heat capacity of PCC  Heat capacity of PEG  Electrical conductivity  Volume fraction  Critical volume fraction  Dimensional constant |
| --- | --- |

**Table S2** Important abbreviations used in this work

| Abbreviation | Description |
| --- | --- |
| LIBs | Lithium-ion batteries |
| PCL | Polycaprolactone |
| PEG | Polyethylene glycol |
| PCC | Phase change composites |
| PCC1000 | Phase change composites 1000 |
| PCC1500 | Phase change composites 1500 |
| PCMs | Phase change materials |
| PTC | Positive temperature coefficient |
| SOC | State of charge |
| SHMs | Sensible heat materials |
| TCR | Temperature coefficient of resistance |
| LHMs | Latent heat materials |
| GNPs | Graphene nanoplatelets |
| CNTs | Carbon nanotubes |

**
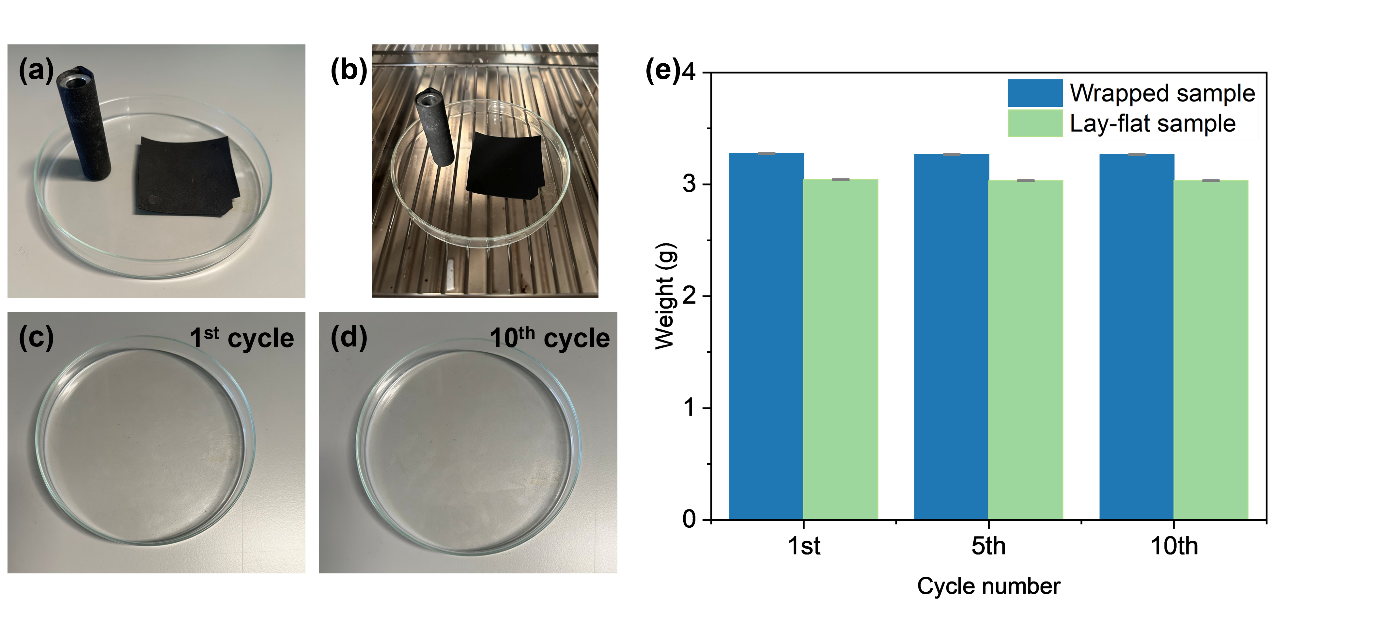
**

**Figure S1** (a) The leakage proof testing set-up with one sample wrapping up the dummy cell and one sample lay flat; (b) The testing condition in ovens from 20 °C to 80 °C; (c) and (d) The appearance of the holding petri dish before and after cyclability tests; (e) The weight difference of wrapped and lay-flat samples over 10 cycles

**
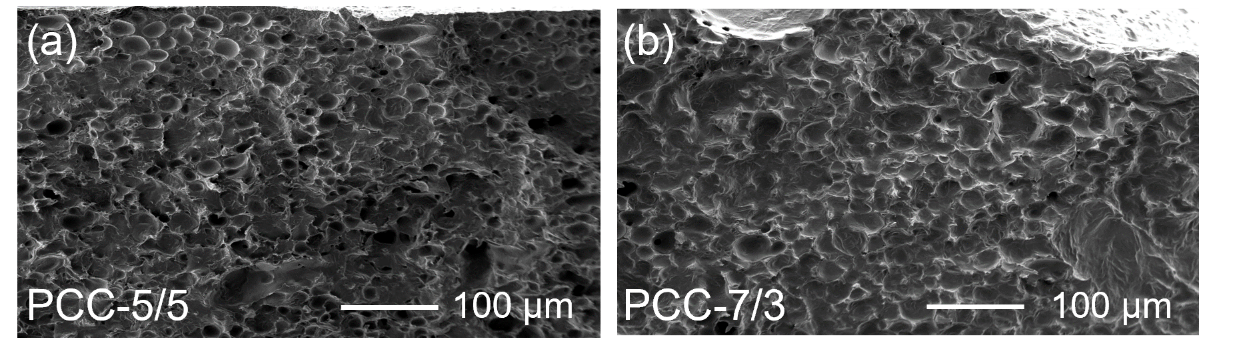
**

**Figure S2** The morphology of (a) PCC1500 at 5:5 ratio between PEG1500 and PCL, and (b) PCC1500 at 7:3 ratio between PEG1500 and PCL.

**
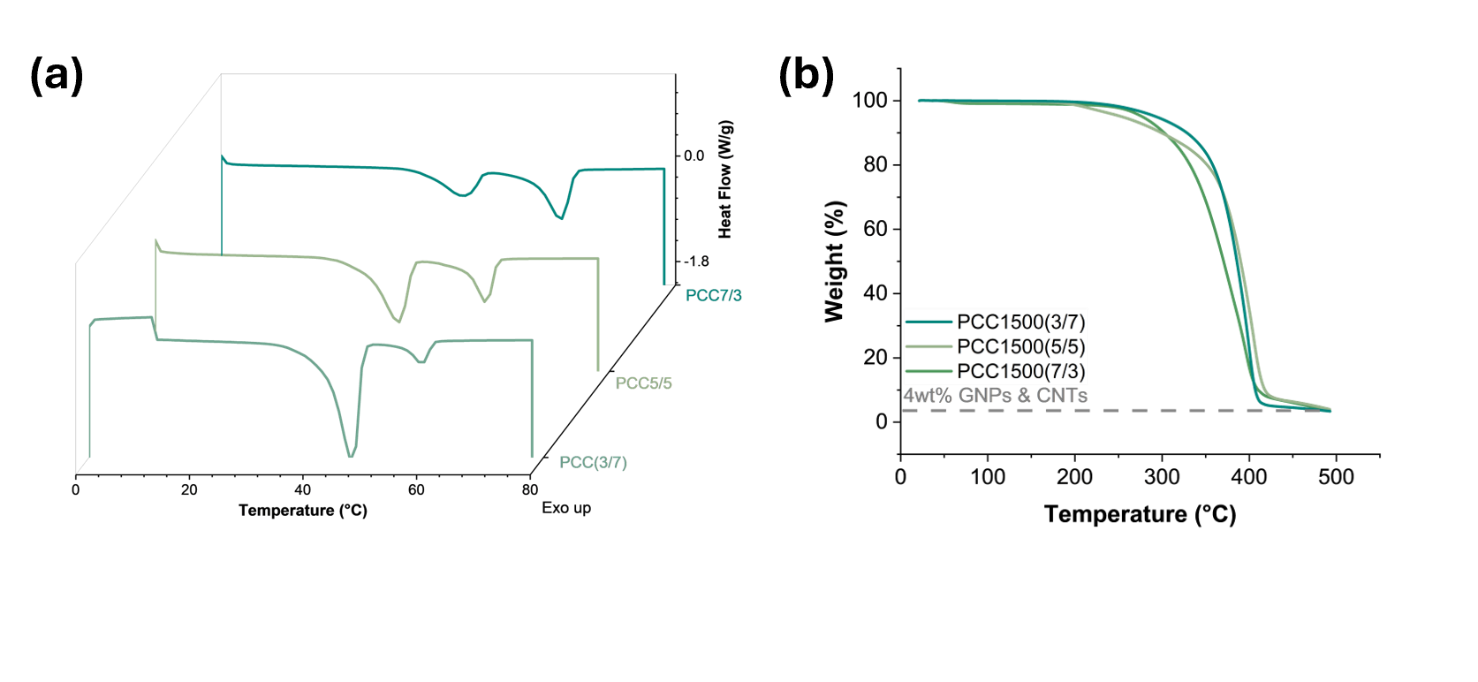
**

**Figure S3** (a) and (b) Latent heat capacity and thermal stability of PCC1500 at various ratios between PEG1500 and PCL.


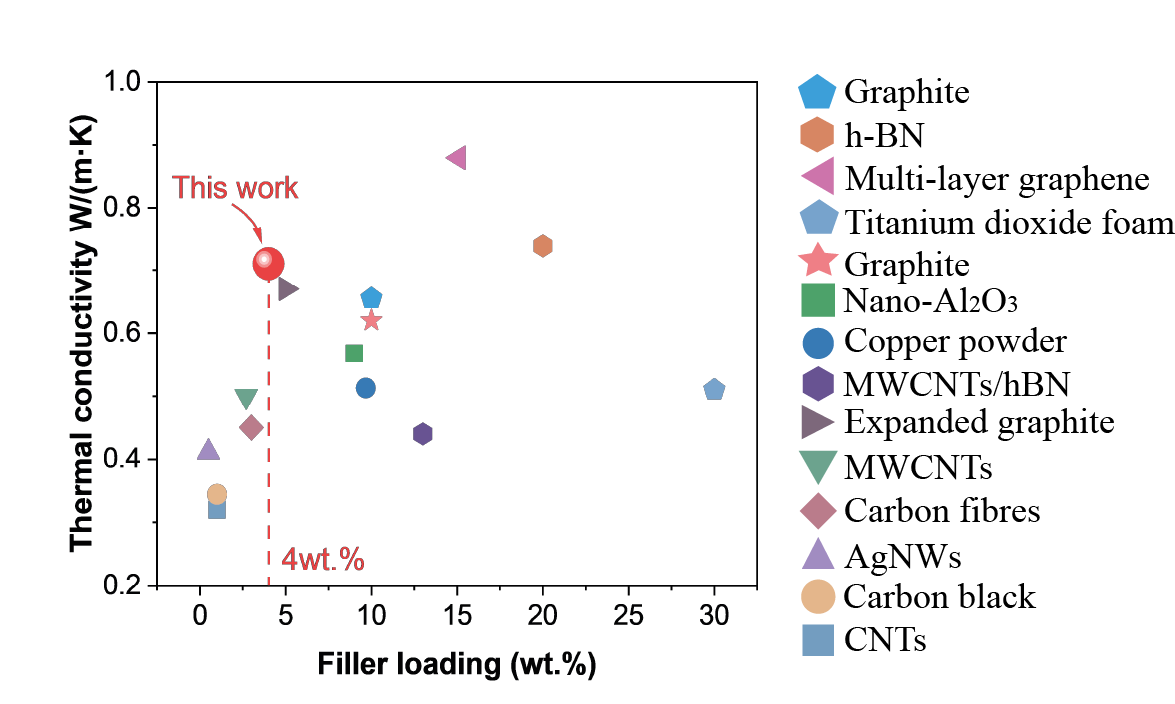


**Figure S4** The comparison in thermal conductivity of multiple fillers incorporated PCMs at various loading, data resourced from ref: [1–13] (From Graphite to CNTs).

**Table S3** Detailed information in DSC spectrums of the materials

| Material | Phase change behaviour | | Melting point (°C) | Heat capacity (J/g) |
| --- | --- | --- | --- | --- |
|  | Onset (°C) | End (°C) |  |  |
| PCL | 31.49 | 66.52 | 59.90 | 61.97 |
| PEG1500 | 30.13 | 48.53 | 44.23 | 208.4 |
| PEG1000 | 19.87 | 36.74 | 31.04 | 150.1 |
| PCC1500(7/3) | 30.24 | 48.15 | 44.57 | 29.17 |
| PCC1500(5/5) | 29.72 | 47.66 | 42.92 | 72.90 |
| PCC1500(3/7) | 32.39 | 50.12 | 46.26 | 129.07 |
| PCC1000(3/7) | 18.75 | 38.27 | 33.00 | 97.50 |


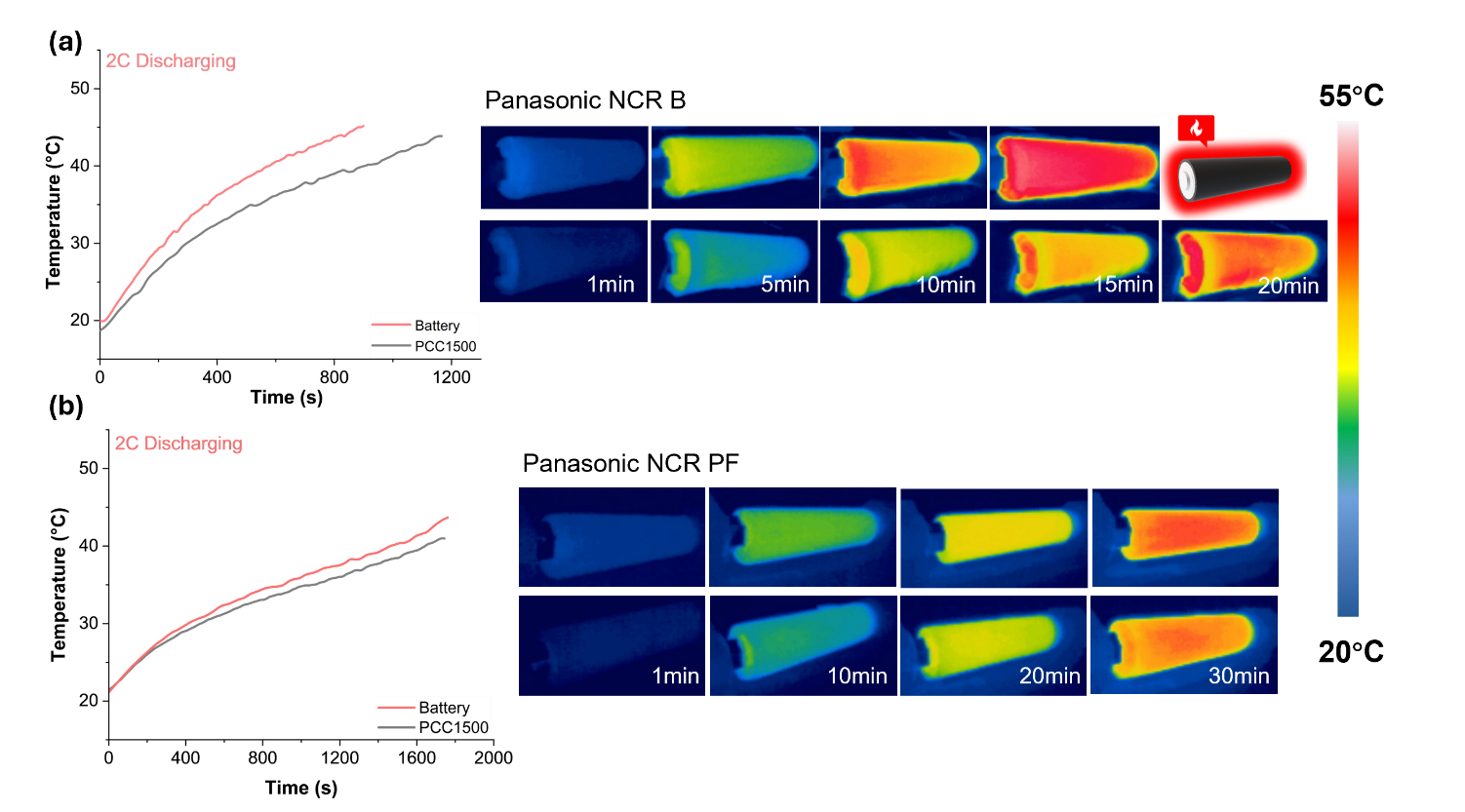


**Figure S5** (a) The passive cooling performance of PCC1500 at 2C discharging rate, tested on Panasonic NCR B cell (3.35 Ah), (b) The passive cooling performance of PCC1500 at 2C discharging rate, tested on Panasonic NCR PF cell (2.9 Ah)


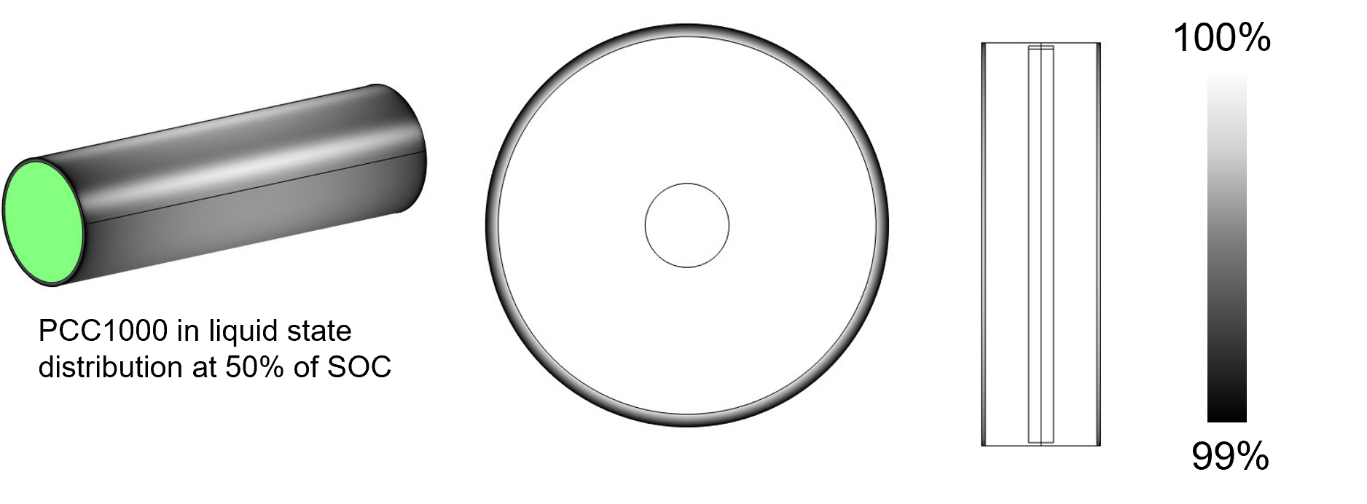


**Figure S6** At 2C discharge rate, the distribution of liquid PCC1000 at 50% of SOC and its horizontal and vertical cross-sectional distribution

**Battery Modelling**

The battery model is established based on Lumped Single Particle Model (LSPM), proposed by Ekström et al[14]. LSPM assumes that only one electrode contributes to diffusion-related voltage losses at the cell level, enabling a replacement of two electrode particles in the original SPM with a single particle.

The total overpotential is pertinent to ohmic, activation and concentration overpotential, relating to the $Q_{irrev}$, defined by Equation S1:

$\eta=\eta_{ohm}+\eta_{act}+\eta_{conc}$ (S1)

where the $\eta_{ohm}$ is due to ohmic resistance (R_ohm_) in the movement of ions and electrons, as described by Ohm’s law in Equation S2:

$\eta_{ohm}=R_{ohm}I_{batt}=\frac{\eta_{ohm}, 1C}{I_{1C}}I_{batt}$ (S2)

where $I_{batt}$ denotes the applied current, $I_{1C}$ is the current at 1C rate, $\eta_{ohm}, 1C$ is the ohmic overpotential at 1C rate.

The activation overpotential ($\eta_{act}$) reflects the activation barrier in electrochemical reactions taking place in the cell, defined by Equation S3:

$\eta_{act}=\frac{2RT}{F}arcsinh(\frac{I_{batt}}{2J_{0}I_{1C}})$ (S3)

where R is the molar ideal gas constant, F is the Faraday constant, T represents the absolute temperature of the cell, $J_{0}$ is the dimensionless charge exchange current rate.

The concentration overpotential ($\eta_{conc}$) resulting from the finite transport rates of reacting material within the cell, can be calculated by can be calculated by applying Fick’s diffusion equation to a dimensionless local particle SOC (S) over a one-dimensional particle length of 1. The equation incorporates a dimensionless spatial variable X, which varies from 1 (particle surface) to 0 (particle centre), defined by Equation S4:

$\tau\frac{dS}{dt}=\nabla^{2}S$ (S4)

where $\tau$ is the diffusion time constant. Thew boundary conditions are given by the Equation S5:

${\nabla S}_{X=o}=-\frac{\tau I_{batt}}{N_{shape}Q_{batt,0}}$ (S5)

where $N_{shape}$ is 3 for the spherical coordinate in this study, $Q_{batt,0}$ is the initial cell capacity.

The battery average SOC (SOC_ave_) is calculated by integrating over the volume of the particles, given in Equation S6:

${SOC}_{ave}=3\int_{0}^{1} S^{2}dX$ (S6)

Therefore, the $\eta_{conc}$ is defined in Equation S7:

$\eta_{conc}=E_{ocv}\left( S_{X=1},T \right)-E_{ocv}\left( {SOC}_{ave},T \right)$ (S7)

The entropy change of the cell is given by the Equation S8:

$E_{entropy}=(T-T_{ref})\frac{\partial E_{ocv}({SOC}_{ave})}{\partial T}$ (S8)

where the entropy coefficient of the Panasonic NCR18650PF cell is retrieved from the study conducted by Lu et al.[15], listed in Table S4.

Subsequently, the OCV under various temperatures (E_OCV_(SOC_ave_,T)) is the sum of reference OCV (E_OCV, ref_(SOC_ave_)) and the entropy voltage (E_entropy_) change, as shown in Equation S9:

$\left( E_{OCV}\left( {SOC}_{ave},T \right) \right)= E_{OCV, ref}\left( {SOC}_{ave} \right)+E_{entropy}$ (S9)

Due to the temperature-sensitive nature, the electrochemical parameters ($\eta_{ohm}$, $J_{0}$, and $\tau$) can be modelled by coupling the Arrhenius dependency[14]. Arrhenius dependency relates these parameters at present temperature to those at a reference temperature (i.e. at which the thermal management tests were conducted) via an exponential function with activation energy $E_{act}$, represented by Equation S10, 11, and 12:

$\eta_{ohm}=\eta_{ohm,1C,ref} exp \left[ \frac{E_{act}^{\eta_{ohm,1C}}}{R}\left( \frac{1}{T}-\frac{1}{T_{ref}} \right) \right]$ (S10)

$J_{0}=J_{0,ref} exp\left[ \frac{E_{act}^{J_{0}}}{R}\left( \frac{1}{T}-\frac{1}{T_{ref}} \right) \right]$ (S11)

$\tau= \tau_{ref} exp\left[ \frac{E_{act}^{\tau}}{R}\left( \frac{1}{T}-\frac{1}{T_{ref}} \right) \right]$ (S12)

**Heat transfer model**

The heat model of working temperatures of the battery cell with and without the application of PCCs was determined by the Bernardi’s heat generation model[16]. The total heat generation rate from cell consists of the irreversible heat and the reversible heat, as shown in Equation S13:

$Q_{total}=Q_{irrev}+Q_{rev}$ (S13)

Theoretically, $Q_{irrev}$ is caused by: 1) Ohmic losses ($Q_{\eta_{ohm}}$) in electrodes, electrolyte, and current collectors, 2) activation losses ${(Q}_{\eta_{act}})$ for the charge transfer reactions, and 3) concentration losses ($Q_{mix}$) resulting from the concentration gradients that generate heat due to non-ideal heat of mixing, given by the Equation S14:

$Q_{irrev}=Q_{\eta_{ohm}}+Q_{\eta_{act}}+Q_{mix}$ (S14)

where:

$Q_{\eta_{ohm}}=\eta_{ohm}I_{batt}$ (S15)

$Q_{\eta_{act}}=\eta_{act}I_{batt}$ (S16)

$Q_{mix}=\frac{N_{shape}Q_{cell,0}}{\tau_{arrh}}\int_{0}^{1} \frac{\partial E_{OCV,therm}}{\partial S}\frac{\partial^{2}S}{\partial^{2}X}X^{N_{shape}-1}\partial X$ (S17)

And the $Q_{rev}$is caused by the entropy changes in the electrode reactions, given by the Equation S18:

$Q_{mix}=T\frac{\partial E_{OCV}(S_{x=1})}{\partial T}I_{batt}$ (S18)

The parameters used of the PCCs developed for establishing the heat model and the specifications of the Panasonic NCR18650PF cell are listed in Table S5 and Table S6, respectively.

**PCMs model**

Assuming no mixing between the liquid and solid phase during the phase change processes, the PCMs model can be established based on a model proposed by Kylili et al., as shown in Equation S19[17]:

$\rho C_{eq}\frac{\partial T}{\partial t}+ \nabla\cdot\left( -K_{eq}\nabla T \right)=Q-Q_{air}$ (S19)

where $\rho$, $C_{eq}$, and $K_{eq}$ denote the density, effective heat capacity at constant pressure, and effective thermal conductivity, respectively. $T$ is the temperature, $Q$ is the heat generated by the battery cell, and the $Q_{air}$ is the heat dissipated by air, defined in Equation S20:

$Q_{air}=h(T-T_{amb})$ (S20)

where $h$ and $T_{amb}$ represent the convective heat transfer coefficient (W m^-2^ K^-1^) and ambient temperature (K) respectively.

When the PCC reaches its phase change temperature $T_{m}$, an assumption has been made that the phase change takes place throughout a period. This time interval is defined between $T_{m}-\Delta T/2$ and $T_{m}+\Delta T/2$, while the phase of the composites during this period is defined by the function $\theta$, representing the fraction of phase before the phase change, defined at below conditions:

$$\theta=1, before T_{m}-\Delta T/2$$

$$\theta=0, after T_{m}+\Delta T/2$$

Accordingly, the density ($\rho$) and specific enthalpy ($H$) are expressed by Equation S21 and S22:

$\rho=\theta\rho_{phase1}+(1-\theta)\rho_{phase2}$ (S21)

$\rho H=\theta\rho_{phase1}H_{phase1}+(1-\theta)\rho_{phase2}H_{phase2}$ (S22)

where $phase1$ and $phase2$ indicate the PCMs in solid phase or in liquid phase, respectively. The specific heat capacity can then be expressed in Equation S23:

$C_{p}=\frac{1}{\rho}\left( \theta_{1}\rho_{phase1}C_{p,phase1}+\theta_{2}\rho_{phase2}C_{p,phase2} \right)+(H_{phase2}-H_{phase1})\frac{da_{m}}{dT}$ (S23)

where

$\theta_{1}=\theta$ and $\theta_{2}=1-\theta$

The mass fraction and the specific heat capacity is defined in Equation S24 and S25:

$a_{m}=\frac{1}{2}\frac{\theta_{2}\rho_{phase2}-\theta_{1}\rho_{phase1}}{\rho}$ (S24)

$C_{eq}=\frac{1}{2}(\theta_{1}\rho_{phase1}C_{p,phase1}+\theta_{2}\rho_{phase2}C_{p,phase2})$ (S25)

And the distribution of latent heat is defined in Equation S26:

$C_{L}\left( T \right)=(H_{phase2}-H_{phase1})\frac{dam}{dT}$ (S26)

$C_{L}$ is then approximated such as the total heat per unit volume released during the phase change equals with the latent heat, L, denoted in Equation S27:

$\int_{T_{pc}-\frac{\Delta T}{2}}^{T_{pc}+\frac{\Delta T}{2}} C_{L}\left( T \right)dT=L\int_{T_{pc}-\frac{\Delta T}{2}}^{T_{pc}+\frac{\Delta T}{2}} \frac{dam}{dT}dT=L$ (S27)

Accordingly, the apparent heat capacity, the effective thermal conductivity and the effective density are defined in Equation S28, S29, and S30:

$C_{p}=\frac{1}{\rho}\left( \theta_{1}\rho_{phase1}C_{p,phase1}+\theta_{2}\rho_{phase2}C_{p,phase2} \right)+C_{L}$ (S28)

$k=\theta_{1}k_{phase1}+\theta_{2}k_{phase2}$ (S29)

$\rho=\theta_{1}\rho_{phase1}+\theta_{2}\rho_{phase2}$ (S30)

**Table S4** Variation of the entropy coefficient with SOC[15]

| ${SOC}_{ave}$ | $\frac{{\partial E}_{OCV, ref}\left( {SOC}_{ave} \right)}{\partial T}$ (mV K^-1^) |
| --- | --- |
| 0.9 | 0.083 |
| 0.8 | 0.083 |
| 0.7 | 0.065 |
| 0.6 | 0.143 |
| 0.5 | 0.169 |
| 0.4 | 0.141 |
| 0.3 | 0.058 |
| 0.2 | -0.082 |
| 0.1 | 0.069 |

**Table S5** Parameters used for battery model establishment

| $\eta_{ohm,1C}$ (mV) | 51.7 |
| --- | --- |
| $J_{0}$ (-) | 0.3 |
| $\tau$ (s) | 6712 |
| $E_{act}^{\eta_{ohm,1C}}$ (kJ mol^-1^) | 24.36 |
| $E_{act}^{J_{0}}$ (kJ mol^-1^) | -58.98 |
| $E_{act}^{\tau}$ (kJ mol^-1^) | 18.58 |

**Table S6** Parameters used for PCMs model establishment

|  | PCC1500 | PCC1000 |  |
| --- | --- | --- | --- |
| Parameters | Value | Value | Unit |
| Phase change temperature | 32.39-50.12 | 18.75-38.27 | °C |
| Latent heat capacity | 129.9 | 93.5 | J/g |
| Thermal conductivity | 0.71 | 0.71 | W/mK |
| Density | 1.18 | 1.18 | g/cm^3^ |
| Heat capacity | 2.47 [18] | 2.14 [19] | J/g |

**Table S7** Specifications of the Panasonic NCR18650PF battery cell

| Electrodes composition | Li(NiCoAl)O_2_/Carbon |
| --- | --- |
| Capacity | 2.9 Ah |
| Min/Max voltage | 2.5 V / 4.2 V |
| Charge temperature | 0 – 45 °C |
| Discharge temperature | -20 – 60 °C |
| Storage temperature | -20 – 50 °C |

**Table S8** Specifications of the Panasonic NCR18650B battery cell

| Electrodes composition | Li(NiCoAl)O_2_/Carbon |
| --- | --- |
| Capacity | 3.35 Ah |
| Min/Max voltage | 2.5 V / 4.2 V |
| Charge temperature | 0 – 45 °C |
| Discharge temperature | -20 – 60 °C |
| Storage temperature | -20 – 50 °C |

**Reference**

1. Li, C., Li, Q., Ge, R.: Synthesis and investigation of form-stable myristic acid based composite phase change material containing styrene ethylene butylene styrene with enhanced properties for thermal energy storage. Journal of Energy Storage. 52, 104594 (2022). https://doi.org/10.1016/J.EST.2022.104594

2. Marske, F., Lindenberg, T., Martins de Souza e Silva, J., Wehrspohn, R.B., Maijenburg, A.W., Hahn, T., Enke, D.: Size and surface effects of hexagonal boron nitrides on the physicochemical properties of monolithic phase change materials synthesized via sol–gel route. Appl Therm Eng. 196, 117325 (2021). https://doi.org/10.1016/J.APPLTHERMALENG.2021.117325

3. Huang, Z., Wang, C., Zhou, L., Wu, C.: Thermal conductivity enhancement and shape stability of phase-change materials using high-strength 3D graphene skeleton. Surfaces and Interfaces. 26, 101338 (2021). https://doi.org/10.1016/J.SURFIN.2021.101338

4. Zhong, Y., He, X., Wang, W., Xu, Y., Wang, Y., Shuai, Y.: Performance enhancement of graphite-based flexible composite phase change materials and heat dissipation characteristics of electronic devices. Applied Thermal Engineering. 263, 125393 (2025). https://doi.org/10.1016/J.APPLTHERMALENG.2024.125393

5. Zhao, Z., Chen, G., Liu, X., Shao, L., Xu, Y., Zhang, X., Li, Y., Yan, Z., Zou, T.: Minireview on Design of Flexible Composite Phase Change Materials to Various Energy Applications: Progresses and Perspectives. Energy and Fuels. 37, 6348–6364 (2023). https://doi.org/10.1021/ACS.ENERGYFUELS.3C00266

6. Zhang, X., Huang, Z., Ma, B., Wen, R., Zhang, M., Huang, Y., Fang, M., Liu, Y.G., Wu, X.: Polyethylene glycol/Cu/SiO 2 form stable composite phase change materials: preparation, characterization, and thermal conductivity enhancement. RSC Adv. 6, 58740–58748 (2016). https://doi.org/10.1039/C6RA12890D

7. Ma, Y., Wang, H., Zhang, L., Sheng, X., Chen, Y.: Flexible phase change composite films with improved thermal conductivity and superb thermal reliability for electronic chip thermal management. Composites Part A: Applied Science and Manufacturing. 163, 107203 (2022). https://doi.org/10.1016/J.COMPOSITESA.2022.107203

8. Mishra, D.K., Bhowmik, S., Pandey, K.M.: Development and Assessment of Beeswax/Expanded Graphite Composite Phase Change Material for Thermal Energy Storage. Arabian Journal for Science and Engineering. 47, 8985–9004 (2022). https://doi.org/10.1007/S13369-021-06476-9

9. Cheng, P., Gao, H., Chen, X., Chen, Y., Han, M., Xing, L., Liu, P., Wang, G.: Flexible monolithic phase change material based on carbon nanotubes/chitosan/poly(vinyl alcohol). Chemical Engineering Journal. 397, 125330 (2020). https://doi.org/10.1016/J.CEJ.2020.125330

10. Liu, Z., Wei, H., Tang, B., Xu, S., Shufen, Z.: Novel light–driven CF/PEG/SiO2 composite phase change materials with high thermal conductivity. Solar Energy Materials and Solar Cells. 174, 538–544 (2018). https://doi.org/10.1016/J.SOLMAT.2017.09.045

11. Li, Y., Chen, Y., Huang, X., Jiang, S., Wang, G.: Anisotropy-functionalized cellulose-based phase change materials with reinforced solar-thermal energy conversion and storage capacity. Chemical Engineering Journal. 415, 129086 (2021). https://doi.org/10.1016/J.CEJ.2021.129086

12. Colla, L., Fedele, L., Mancin, S., Danza, L., Manca, O.: Nano-PCMs for enhanced energy storage and passive cooling applications. Appl Therm Eng. 110, 584–589 (2017). https://doi.org/10.1016/J.APPLTHERMALENG.2016.03.161

13. Karaipekli, A., Biçer, A., Sarı, A., Tyagi, V.V.: Thermal characteristics of expanded perlite/paraffin composite phase change material with enhanced thermal conductivity using carbon nanotubes. Energy Conversion and Management. 134, 373–381 (2017). https://doi.org/10.1016/J.ENCONMAN.2016.12.053

14. Ekström, H., Fridholm, B., Lindbergh, G.: Comparison of lumped diffusion models for voltage prediction of a lithium-ion battery cell during dynamic loads. Journal of Power Sources. 402, 296–300 (2018). https://doi.org/10.1016/J.JPOWSOUR.2018.09.020

15. Lu, Z., Yu, X.L., Wei, L.C., Cao, F., Zhang, L.Y., Meng, X.Z., Jin, L.W.: A comprehensive experimental study on temperature-dependent performance of lithium-ion battery. Applied Thermal Engineering. 158, 113800 (2019). https://doi.org/10.1016/J.APPLTHERMALENG.2019.113800

16. Bernardi, D., Pawlikowski, E., Newman, J.: GENERAL ENERGY BALANCE FOR BATTERY SYSTEMS. Electrochemical Society Extended Abstracts. 84–2, 164–165 (1984). https://doi.org/10.1149/1.2113792/XML

17. Numerical Heat Transfer Analysis of a Phase Change Material (PCM) - Enhanced Plaster, https://www.comsol.com/paper/numerical-heat-transfer-analysis-of-a-phase-change-material-pcm-enhanced-plaster-39851

18. Azizi, Y., Sadrameli, S.M.: Thermal management of a LiFePO4 battery pack at high temperature environment using a composite of phase change materials and aluminum wire mesh plates. Energy Conversion and Management. 128, 294–302 (2016). https://doi.org/10.1016/J.ENCONMAN.2016.09.081

19. Kou, Y., Wang, S., Luo, J., Sun, K., Zhang, J., Tan, Z., Shi, Q.: Thermal analysis and heat capacity study of polyethylene glycol (PEG) phase change materials for thermal energy storage applications. The Journal of Chemical Thermodynamics. 128, 259–274 (2019). https://doi.org/10.1016/J.JCT.2018.08.031
